# Preliminary development of a questionnaire to measure the extra-pulmonary symptoms of severe asthma

**DOI:** 10.1186/s12890-021-01730-0

**Published:** 2021-11-14

**Authors:** Giulio de Felice, Michael E. Hyland, Joseph W. Lanario, Yuri Antonacci, Rupert C. Jones, Matthew Masoli

**Affiliations:** 1 Sapienza University of Rome, Rome, Italy; 2 NC IUL University, London, UK; 3 Plymouth Marjon University, Plymouth, UK; 4 Plymouth University, Plymouth, UK; 5 University of Palermo, Palermo, Italy; 6 Royal Devon and Exeter Hospital, Exeter, UK; 7 University of Exeter, Exeter, UK

**Keywords:** Asthma, Symptoms, Fibromyalgia, Questionnaire, Patient reported outcomes

## Abstract

**Background:**

Research into the effects of asthma treatments on the extra-pulmonary symptoms of severe asthma is limited by the absence of a suitable questionnaire. The aim was to create a questionnaire suitable for intervention studies by selecting symptoms that are statistically associated with asthma pathology and therefore may improve when pathology is reduced.

**Methods:**

Patients attending a specialist asthma clinic completed the 65-item General Symptom Questionnaire (GSQ-65), a questionnaire validated for assessing symptoms of people with multiple medically unexplained symptoms. Lung function (FEV1%) and cumulative oral corticosteroids (OCS) calculated from maintenance dose plus exacerbations were obtained from clinic records. Pathology was represented by the two components of a principal component analysis (PCA) of FEV1% and OCS. LASSO regression was used to select symptoms that had high coefficients with these two principal components and occurred frequently in severe asthma.

**Results:**

100 patients provided data. PCA revealed two components, one where FEV1% and OCS were inversely related and another where they were directly related. LASSO regression revealed 39 symptoms with non-zero coefficients on one or more of the two principal components from which 16 symptoms were selected for the GSQ-A on the basis of magnitude of coefficient and frequency. Asthma symptoms measured by asthma control questionnaires were excluded. The GSQ-A correlated 0.33 and − 0.34 (*p* = 0.001) with the two principal components.

**Conclusion:**

The GSQ-A assesses the frequency of 16 heterogenous non-respiratory symptoms that are associated with asthma severity using the statistical combination of FEV1% and OCS.

**Supplementary Information:**

The online version contains supplementary material available at 10.1186/s12890-021-01730-0.

## Background

The experience of people with severe asthma differs in several ways compared to those with mild or moderate asthma including extra-pulmonary symptoms (i.e., symptoms beyond the lung). There are two possible aetiologies. First, there are those symptoms that have no causal relationship with the underlying lung pathology, including the symptoms of unrelated co-morbidities and symptoms resulting from the side effects of treatment. Second, there are those that have some form of direct or indirect causal connection with asthma pathology, mediated, for example, through the interaction between specific and non-specific inflammatory mediators.

All extra-pulmonary symptoms are relevant to clinical practice and, because these symptoms are very varied, can be measured by any of the several general symptom questionnaires that have been developed for other purposes such as the 164-item COMPASS [1] or its shorter 31-item version the COMPASS-31 [2] designed to assess neurological symptoms, the PILL designed for the general population [3] or the General Symptom Questionnaire (GSQ-65) designed to assess the symptomatology of people with a range of medically unexplained symptoms that are associated with a combination of neurological, immune and endocrine abnormalities [4]. Although these questionnaires cover a wide range of different symptoms and can be useful in clinical practice, they are long and not optimised for studies of interventions for the treatment of asthma which directly target asthma pathology

Pharmacological and non-pharmacological interventions that reduce asthma pathology should reduce those extra-pulmonary symptoms that are causally influenced by asthma pathology. If a general-purpose symptoms questionnaire is used in a clinical trial, then the scale may include items that are not improved by pathology reduction. Additionally, it is unclear whether any reduction in extra-pulmonary symptoms is due to the cessation of treatments that cause side effects (e.g., oral corticosteroids) or due to the pathology reducing effect of the treatment itself.

The aim of this study was to create a preliminary severe asthma specific extra-pulmonary questionnaire by selecting extra-pulmonary symptoms from a generic symptom questionnaire. The General Symptom Quesitonnaire-65 (GSQ-65) was designed to assess the varied and overlapping symptoms of people with medically unexplained symptoms, including people diagnosed with fibromyalgia, chronic fatigue syndrome (CFS) and irritable bowel syndrome (IBS). Common comorbidities of asthma include fibromyalgia [5–7], IBS [5, 8–10], and gastroesophageal disease (GORD) [11, 12].

There are two ways of selecting symptoms from the GSQ-65 for use in asthma clinical trials. One is to use the full scale in a clinical trial and then select those symptoms that are most sensitive to change. The disadvantage of this method is that item selections would be based on response to a particular treatment. As different treatments can affect extra-pulmonary symptoms in different ways, any resulting questionnaire will be biased towards the treatment that was used in its derivation. We used an alternative method, which is to select items that are related to asthma pathology, as this method produces a selection of symptoms that is unbiased towards any particular treatment. In this study we took lung function as the primary indicator of asthma pathology and cumulative oral corticosteroid (OCS) use over 12 months as a secondary indicator calculated from exacerbations and maintenance dose. We selected symptoms that vary with lung function and cumulative OCS but also restricted the symptoms to those whose frequency and other properties make them suitable for use in a clinical trial.

## Methods

### Participants

Patients diagnosed with severe asthma as defined by guidelines [13] took part in the study. Inclusions criterion was aged ≥ 16 years and exclusion criterion was another condition that could contribute significantly to symptoms.

### Questionnaire

*General symptom questionnaire *(*GSQ*). The 65-item questionnaire assesses the frequency of somatic and psychological symptoms on a 6 point Likert scale (the value scoring for each response shown in brackets): “Never or almost never” (1), “Less than 3 or 4 times per year” (2), “Every month or so” (3), “Every week or so” (4), “More than once per week” (5) or “Every day” (6) [4].

### Clinic data

We obtained two measures of asthma severity from clinic records, (a) percentage predicted forced expiratory volume in the first second (FEV1%) and (b) cumulative oral corticosteroids (OCS) exposure (mg/year). OCS dose was calculated by combining the daily dose and estimated dose for exacerbations following GINA guidelines [14] equating to 280 mg of OCS per exacerbation.

### Procedure

Written informed consent was obtained after which participants completed the questionnaire either at home or during their clinic visit. The questionnaire data were collected at the same time as other questionnaires data were collected for a study reported elsewhere [15].

### Statistics

The reduction of the 65 item set of the GSQ-65 was conducted in three stages. In Stage 1, one of any pair of items correlating at > 0.7 was removed so that the selected items contributed different variation to the overall score. The item of the pair with the lowest frequency of occurrence was removed. In Stage 2 items were removed if they had a minimal relationship with pathology. Pathology was assessed by pulmonary function (FEV1%) and cumulative oral corticosteroid dose (OCS). High FEV1% and low OCS indicate low pathology, but the two variables are causally linked as treatment with OCS increases FEV1%. Therefore, in order to provide a combined measure of pathology, OCS and FEV1% were entered into a principal component analysis (PCA). The resulting two components (PC1 and PC2) represent the combination of OCS and FEV1% and therefore a better representation of pathology than separate use of the two variables [16, 17]. PC1 and PC2 were then used as the dependent variables and the remaining GSQ-65 items as independent variables in a penalized linear regression that was solved by applying the Least Absolute Shrinkage and Selection Operator (LASSO) on the whole sample. LASSO is a regression analysis method that performs both variable selection and regularization in order to enhance the prediction accuracy and interpretability of the statistical model [18]. The trade-off between interpretability and prediction accuracy is controlled by a shrinkage parameter that is estimated through a cross-validation procedure after the standardization of both dependent and independent variables [19]. In the present analysis, LASSO checks the association between symptoms (items of the GSQ-65) and pathology (PC1 and PC2), assigns a weight to each symptom and eliminates those symptoms that are poorly associated with pathology by assigning a zero coefficient. The symptoms that still have a non-zero coefficient are then selected to be part of the model.

LASSO regression has advantages over other methods of item selection particularly when there are large numbers of independent variables which may be even more numerous than the observations [20]. In such conditions, the presence of strongly correlated independent variables is overcome by excluding the least associated with the dependent variable [21]. Thus, LASSO regression eliminates irrelevant symptoms early on and therefore makes interpretation easier. Built on linear modeling, LASSO tries to find the relationship between dependent and independent variables where the coefficients correspond to the amount of expected change in the dependent variable for a unit increase/decrease in the independent variable (this is why in the output there are positive and negative coefficients). The standardization of independent variables guarantees that the coefficients are not selected because of their scale but rather their relative importance [22].

At Stage 2, all items with zero coefficients from the LASSO regression on both principal components were removed. At Stage 3, items from those remaining were selected on the basis of their coefficients, frequency of occurrence and clinical properties.

Correlations with the GSQ-A are Pearson correlations. Internal consistency was assessed by Cronbach’s alpha.

### Ethics

The study, as well as questionnaire data collection and access to patient records, was approved by the University Hospitals Plymouth NHS Trust and the Newcastle & North Tyneside Research Ethics Committee and the Newcastle & North Tyneside Health Research Authority (ethical approval number 16/NE/0188, IRAS ID: 207601).

## Results

Completed questionnaires were received from 100 patients, mean age (SD) = 54 (15) years 63% were female. Sample characteristics are shown in Table 1.

**Table 1:** Baseline characteristics* if study population.

Total sample = 100	63 females, 37 males
Age, mean (range)	54.19–81
FEV1% predicted	69.7 (66.1–73.4)
BMI	31.6 (29.9–33.3)
Number of severe exacerbations in the previous 12 months	2.1 (1.4–2.7)
Hospital admissions in the previous 12 months	0.3 (0–0.8)
Number (percent) on maintenance oral corticosteroids	38 (38%)
Number (percent) on biologic therapy	32 (32%)

*Mean values (confidence intervals) of sample unless stated otherwise

Three items were removed at Stage 1 because of high inter-correlations with other items leaving 62 items. Stage 2 requires a measure of pathology to be constructed from the combination of FEV1% and cumulative OCS. When these two variables were entered into a principal component analysis, the first component accounted for 64% of the variance. In PC1 FEV1% loaded 0.80 and cumulative OCS loaded − 0.8. Thus, this component indicates healthy participants with comparatively good pulmonary function with comparatively low requirement for OCS: it is an inverse measure of the severity of pathology. The second component accounted for 36% of the variance. In PC2 FEV1% loaded 0.6 and OCS loaded 0.6. Because both loadings are positive, the second component represents the improvement to FEV1% achieved by OCS, i.e., where comparatively better FEV1% is accompanied by high OCS.

The two measures of pathology (PC1 and PC2) were then used as the two dependent variables in the LASSO regression where 62 items were the independent variables. Table 2 shows the 23 items that have zero coefficients and therefore excluded from further analysis at Stage 2.

**Table 2:** 23 items excluded at Stage 2 of item selection.

Headaches	Irritable
Stomach pain	Jittery easily startled, often worried
Chest pain	More clumsy than others
Back pain	Difficulty getting to sleep
Pain increasing the day after you are active	Blocked nose
Fatigue for no reason	Itchy eyes
Very cold hands or feet	Restless legs
Diarrhoea	Dizziness or loss of balance
Constipation	Cramps in leg foot or bottom
Heartburn	Problems urinating, frequency hesitancy or pain
Depression	Feeling out of breath for no reason
Feeling anxious for no reason	

The coefficients of the remaining 39 items are shown in Table 3. These items all have a plausible relationship with asthma pathology. At Stage 3 a priority list of symptoms was formed by taking all those items with a frequency of 25% or more that *either* had a LASSO coefficient of 0.08 (in absolute value) or more on one component *or* had coefficients of any magnitude on both components. Symptoms with a LASSO coefficient of 0.035 were then added to the priority list if their frequency was greater than 40% per week. From this priority list of 22 items, four items were removed because they were associated with night disturbance or were symptoms linked to atopy. From the remaining 18 items two further items were removed, one (intolerant of some food) because perceived change on this item is likely to be slow and one (difficulty concentrating) was removed because there are two other cognitive items included in the priority list. The symptoms of ‘very vivid dreams’ and ‘nightmares, night terrors’ are correlated 0.53. The former is more than twice as frequent than the latter and makes the criterion of selection into the GSQ-A even though the latter is more strongly associated with pathology. The selection process produces a 16 symptom questionnaire, the GSQ-A, the items of which are shown in Table 3 along with categories indicating why an item was included or excluded.

**Table 3:** Results of LASSO regression with PC1 and PC2 scores as dependent variables, percentage reporting symptom at least once weekly and criteria for symptom selection.

Symptoms	LASSO coefficients with PC1	LASSO coefficients with PC2	% of people reporting at least once per week	Criteria for symptom selection*
Swollen painful joints	0.000	0.011	39	
**Pain moving from one place of body to another on different days**	− 0.087	0.038	34	a1
**Sensitive or tender skin**	− 0.038	0.000	44	a2
**Fatigue increasing the day after you are active**	0.000	0.046	46	a3
Fatigue increasing after a cold or sore throat	0.000	0.020	34	
**Mental fog**	0.100	0.000	46	a4
Difficulty concentrating	0.000	− 0.047	52	ac
**Memory problems**	− 0.101	− 0.090	48	a5
Easily feel too cold	− 0.024	0.000	47	
**Easily feel too hot sweating**	− 0.051	− 0.017	61	a6
Thirsty all the time	0.000	− 0.003	47	
**Bloating of the stomach**	− 0.083	0.000	44	a7
**Nausea for no reason**	− 0.014	0.101	25	a8
Intolerant to some food	0.071	0.036	32	ac
**Ringing in ears**	0.061	0.063	34	a9
**Very vivid dreams**	0.000	− 0.051	40	a10
Nightmares night terrors	0.087	− 0.128	17	
Sensitivity to bright lights	0.012	0.000	26	
Sensitivity to noise	0.000	− 0.033	19	
Waking up often at night	0.000	0.144	69	ab
**Racing heart**	0.000	0.065	43	a11
**Hands tremble or shake**	− 0.091	− 0.027	56	a12
**Face flushes**	0.000	0.074	35	a13
Running nose	0.140	0.000	36	ab
Itchy skin	0.000	− 0.114	51	ab
Head cold sore throat or ‘flu	− 0.116	0.000	6	
Mouth ulcers sores in mouth	0.195	0.012	13	
Skin rash	− 0.034	0.046	17	
Boils or pimples on face or body	− 0.111	0.136	15	
Twitching of eyelid	0.000	0.000	16	
Twitching other than eyelid	0.000	0.109	14	
Feeling faint	0.000	0.142	19	
**Numbness tingling pins and needles**	0.000	− 0.107	44	a14
Loss of voice	0.076	− 0.081	13	
Urinating two or more times per night	− 0.148	0.000	47	ab
**Blurred vision**	− 0.105	0.057	26	a15
Hair loss	0.000	0.196	18	
**Feeling very ill for no reason**	− 0.107	0.000	29	a16

^*^a = Items on priority list; b = Items rejected due to association with asthma; c = items removed for other reasons. 1–16 symptoms to be included in the GSQ-A

The correlation between the GSQ-A and GSQ-65 was 0.97, *p* < 0.001. The GSQ-A correlated with cumulative OCS, 0.46, *p <* 0.001, but not with FEV1%. − 0.06, ns. The GSQ-A correlated − 0.33, *p* = 0.01 with PC1 and 0.34, *p* = 0.001 with PC2. The GSQ-A correlated 0.22 (*p* < 0.05) with BMI. For our sample, the mean of the GSQ-A was 2.06, the standard deviation was 1.2 and each item was scored from 0 to 5.

## Discussion

The aim of this study was to select symptoms from the GSQ-65 that were related to asthma pathology and whose distribution and type increased the likelihood of detecting response to an intervention that reduced asthma pathology. FEV1% and cumulative OCS were used as two interacting measures of pathology and the combination determined by PCA. The PCA provided two components, a primary component where high FEV1% was associated with low OCS, indicative of a general tendency for better health, but also a secondary component where high FEV1% was associated with high OCS. The second component expresses the positive effect that OCS can have on FEV1%.

In order to select items for the new questionnaire, we wanted to exclude items that had a poor relation with asthma pathology, and therefore unlikely to be improved by asthma treatment. Gastro-oesophageal reflux disease is a comorbidity of asthma caused by known mechanisms: it exacerbates asthma due to distal oesophageal stimulation with acid [23–25]. Asthma treatments are therefore unlikely to improve the symptom of heartburn, and though treatment of GORD has the potential for improving asthma symptoms results are mixed [26–28]. In our study, the symptom of heartburn failed to be sufficiently associated with asthma pathology as measured by the LASSO analysis and was rejected at the second stage of item reduction.

Fibromyalgia is a known comorbidity of severe asthma [6] and many symptoms of fibromyalgia were found, by our method, to be related to asthma pathology and were included in the GSQ-A. Like other medically unexplained syndromes, the pathophysiology of fibromyalgia is uncertain because, although there are many biological abnormalities, none are specific, and so diagnosis is based on symptoms. Pain is a primary symptom of fibromyalgia but of the several pain items in the GSQ-65, only one is associated with asthma severity and included in the GSQ-A, namely, ‘pain moving from one place of body to another on different days’. This symptom is highly indicative of central sensitivity syndrome [29] in contrast to the peripheral pain measured by other symptoms in the GSQ-65 and that can have a variety of causes. ‘Sensitive or tender skin’ is a second symptom indicative of central sensitivity syndrome and included in the GSQ-A. Two further symptoms indicative of central sensitivity syndrome (sensitivity to noise and sensitivity to bright lights) have lower coefficients with asthma pathology and so were included at Stage 2 but rejected at Stage 3.

Two cognitive symptoms are included in the GSQ-A, namely ‘mental fog’ which is reported in 91% of people with fibromyalgia and 93% of people with CFS, and ‘memory problems’ which is reported in 90% of people with fibromyalgia and 91% of people with CFS [5]. Although the pathophysiology of fibromyalgia lacks consensus, it is possible that the aetiology of asthma and fibromyalgia are associated due to common inflammatory mechanisms as inflammation is a known pathophysiology of fibromyalgia [30, 31].

Although a comparatively short questionnaire, the symptoms of the GSQ-A are very heterogenous. In addition to two central sensitivity syndrome and two cognitive symptoms, the GSQ-A includes two gastric symptoms, one ocular symptom, one auditory symptom, two thermal regulation symptoms, four neurological symptoms and two symptoms (‘very vivid dreams’ and ‘feeling unwell for no reason’) that could respond quickly to changes in health status. Neither anxiety, depression nor irritability feature in the GSQ-A. These symptoms have a variety causes and therefore the association with asthma pathology was too low to meet the requirement of the LASSO algorithm and so were rejected at Stage 2 of the item reduction process. Despite their heterogeneity, the coefficient of internal consistency was very high at 0.93, confirming the use of the mean of the items as a single scale score.

A symptom can improve at follow up only if it is experienced at baseline. Consequently, a symptom questionnaire is sensitive to change in a given population only if the symptoms assessed are sufficiently common. Patients respond to the GSQ-A on a 6-point scale as they do on the GSQ-65. For ten of the 16 GSQ-A symptoms, 40% of patients reported a frequency of occurrence of either “Every week or so”, “More than once per week”, “Every day” i.e., one of the top three points of the 6-point scale. For a further three symptoms, 30% of patients reported a frequency of occurrence in one of the top three points of the 6-point scale. The questionnaire measures symptoms that vary in frequency but are sufficiently common to be sensitive to improvement in a population of people with severe asthma.

The PCA provided a way of combining FEV1% and cumulative OCS and it did so by producing two different indicators of pathology, PC1 and PC2. Examination of the component loadings shows that FEV1% and cumulative OCS contribute approximately equally to the scores of PC1 and PC2. The GSQ-A mean (i.e. average of scores for each patient) correlated negatively with PC1, showing that healthy participants with better lung function and less requirement for OCS have fewer extra-pulmonary symptoms. However, the GSQ-A mean correlated positively with PC2, showing that participants whose comparatively better FEV1% was achieved by a comparatively high dose of OCS had more extra-pulmonary symptoms. FEV1% did not by itself correlate with either the GSQ-A mean or with the GSQ65 mean, and FEV1% is known to correlate poorly with other patient reported outcomes, so these findings support the use of PCA in creating indices of pathology from multiple causally related indicators of pathology [16, 17].

There are bidirectional causal connections between inflammation in the lung and non-specific inflammatory markers that creates an association between asthma and obesity [32]. The mean BMI of this group of patients was at the bottom of the range normally considered obese and BMI correlated significantly GSQ-A consistent with the hypothesis that the extra-pulmonary symptoms are linked to nonspecific inflammation [33, 34]. Nonspecific inflammation is linked to many other biological abnormalities through a network of biological causal influence [35]. The GSQ-A has been designed for interventions, including both pharmacological and non-pharmacological interventions but use in this way requires validation in a further studies. There are many symptoms that create distress for the patient and measured by the GSQ-65 but not by the GSQ-A. If the aim is to detect the full range of extra-pulmonary symptoms a patient experiences as part of clinical assessment, then other questionnaires would be more appropriate, such as the GSQ-65 [4] which is designed for people with medically unexplained symptoms of varying kinds or the COMPASS [1, 2] which is designed for people with neurological symptoms. Nevertheless, the GSQ-65 and GSQ-A are highly correlated.

### Limitations

This study describes the preliminary development of a questionnaire that requires validation in a larger population. The items for the GSQ-A are a subset of the GSQ-65 and there may be other extra-pulmonary symptoms that are not measured here. Prescription of biologics forms a binary variable and possible for this reason did not contribute additional information about pathology. The data were collected from a single asthma clinic. Data from the GSQ-A is yet to be collected in a clinical trial, the sensitivity of the scale to change is unknown and any such change does not necessarily reflect the impact on quality of life of these symptoms on patients.

## Conclusions

The GSQ-A is a 16-item questionnaire designed to detect change in the extra-pulmonary symptoms of severe asthma resulting from asthma interventions. Symptoms were selected from the GSQ-65 on the basis of their relationship with asthma pathology and are not biased to favour a particular treatment. The questionnaire score is the mean, i.e., adding the scores of the individual items (each item is scored 0–5) and dividing by the total number of items, and following normal practice, one missing item is acceptable, two or more missing (i.e., > 10%) invalidates that patient’s data. The questionnaire is available as a Additional file 1 (GSQ-A).

## Supplementary Information

**Additional file 1.** GSQ-A.

## Data Availability

The data set can be obtained from the first author Giulio de Felice giulio.defelice@uniroma1.it, or from the corresponding author. The questionnaire can be obtained from the corresponding author.

## Abbreviations

BMI: Body mass index
CFS: Chronic fatigue syndrome
FEV1%: Forced expiratory volume in the second expressed as the percentage of that predicted based on sex and height
GORD: Gastro-oesophageal reflux disease
GSQ: General symptom questionnaire
IBS: Irritable bowel syndrome
LASSO: Least Absolute Shrinkage and Selection Operator
OCS: Oral corticosteroids
PCA: Principal component analysis
PC1: First principal component
PC2: Second principal component
